# Respiratory Syncytial Virus and Other Viral Infections among Children under Two Years Old in Southern Vietnam 2009-2010: Clinical Characteristics and Disease Severity

**DOI:** 10.1371/journal.pone.0160606

**Published:** 2016-08-08

**Authors:** Lien Anh Ha Do, Juliet E. Bryant, Anh Tuan Tran, Bach Hue Nguyen, Thi Thu Loan Tran, Quynh Huong Tran, Quoc Bao Vo, Nguyen Anh Tran Dac, Hong Nhien Trinh, Thi Thanh Hai Nguyen, Bao Tinh Le Binh, Khanh Le, Minh Tien Nguyen, Quang Tung Thai, Thanh Vu Vo, Ngoc Quang Minh Ngo, Thi Kim Huyen Dang, Ngoc Huong Cao, Thu Van Tran, Lu Viet Ho, Jeremy Farrar, Menno de Jong, H. Rogier van Doorn

**Affiliations:** 1 Oxford University Clinical Research Unit, Wellcome Trust Major Overseas Program, Ho Chi Minh City, Vietnam; 2 Murdoch Children’s Research Institute, Melbourne, Australia; 3 Nuffield Department of Clinical Medicine, University of Oxford, Oxford, United Kingdom; 4 Children Hospital 1, 341 Su Van Hanh, Ward 10, District 10, Ho Chi Minh City, Vietnam; 5 Children Hospital 2, 14 Ly Tu Trong, Ben Nghe Ward, District 1, Ho Chi Minh City, Vietnam; 6 Department of Medical Microbiology, Academic Medical Center, University of Amsterdam, Amsterdam, The Netherlands; Kliniken der Stadt Köln gGmbH, GERMANY

## Abstract

**Background:**

Despite a high burden of respiratory syncytial virus (RSV) infections among children, data on demographic and clinical characteristics of RSV are scarce in low and middle income countries. This study aims to describe the viral etiologies, the demographic, epidemiological, and clinical characteristics of children under two years of age who were hospitalized with a lower respiratory tract infections (LRTI), focusing on RSV (prevalence, seasonality, subgroups, viral load) and its association with disease severity.

**Methods:**

A prospective study among children under two years of age, hospitalized with LRTI was conducted in two referral pediatric hospitals in Ho Chi Minh City, Vietnam, from May 2009 to December 2010. Socio-demographic, clinical data and nasopharyngeal swabs were collected on enrolment and discharge. Multiplex real-time RT-PCR (13 viruses) and quantitative RSV RT-PCR were used to identify viral pathogens, RSV load and subgroups.

**Results:**

Among 632 cases, 48% were RSV positive. RSV infections occurred at younger age than three other leading viral infections i.e rhinovirus (RV), metapneumovirus (MPV), parainfluenza virus (PIV-3) and were significantly more frequent in the first 6 months of life. Clinical severity score of RSV infection was significantly higher than PIV-3 but not for RV or MPV. In multivariate analysis, RV infection was significantly associated with severity while RSV infection was not. Among RSV infections, neither viral load nor viral co-infections were significantly associated with severity. Young age and having fever at admission were significantly associated with both RSV and LRTI severity. A shift in RSV subgroup predominance was observed during two consecutive rainy seasons but was not associated with severity.

**Conclusion:**

We report etiologies, the epidemiological and clinical characteristics of LRTI among hospitalized children under two years of age and risk factors of RSV and LRTI severity.

## Introduction

Respiratory syncytial virus (RSV) is the leading cause of lower respiratory tract infections (LRTIs) in young children. 50% of children are infected by RSV during their first year of life, and by 3 years of age, 100% have experienced at least one infection [1]. Previous studies [2–4] have shown the importance of RSV in hospitalized children in Vietnam. Hospital records from the two main paediatric referral centers in Ho Chi Minh City show that 77% of hospitalized children with LRTI are under 2 years old (unpublished data). In addition, severe disease from RSV infection seems to exclusively occur in this population [3, 5]. However, information on detailed clinical, epidemiological features and virological characteristics of RSV infections (e.g. disease burden, demographics, seasonal variations of RSV and other viral infections, circulating genotypes and subgroups, viral load) or on the frequency / impact of other respiratory viruses among Vietnamese children under two years old are limited [6].

Here, we aimed to describe the viral etiologies and the demographic, epidemiological, and clinical characteristics of children under two years of age who were hospitalized with a LRTI, focusing on RSV (prevalence, seasonality, subgroups, viral load) and its association with disease severity.

## Materials & Methods

### Study design and study participants

The study was conducted from May 2009 to December 2010 (to cover two RSV seasons, that normally coincide with the rainy season) at Children’s Hospital 1 (CH1) and Children’s Hospital 2 (CH2), the two largest pediatric referral centers for southern Vietnam, located in Ho Chi Minh City. Children from the Respiratory Wards (RW), Emergency Care Units (ECU) and Intensive Care Units (ICU) were eligible for inclusion in the study. Patients admitted to the RW are typically those that initially present to the outpatient clinics and were subsequently indicated for admission, while those admitted to ECU or ICU typically presented with more severe symptoms. Inclusion criteria were age between 1 month to 2 years, cough, a clinical diagnosis of LRTI based on WHO criteria [7], and onset of symptoms ≤ 4 days prior to hospital admission. Exclusion criteria were patients with known non-respiratory or non-infectious respiratory diseases such as asthma.

### Data and samples collection

Socio-demographic data, medical history, clinical data from enrolment to discharge were recorded in standardized case report forms (CRFs). Nasopharyngeal swabs and EDTA blood were collected on the day of enrolment (day 1) and on day 7 or discharge (if patients were discharged before day 7). EDTA blood were used for whole blood cells counting, and liver enzyme measurement by CH1 and CH2 laboratory. Swabs were placed in viral transport medium [8] and kept at 4°C for a maximum of 24h, then aliquoted and stored at -80°C until further processing for molecular diagnostics [9, 10].

### Ethics

The study was approved by the Institutional Review Board of Children’s Hospitals 1 and 2, the Scientific and Ethics Committee of the Hospital for Tropical Diseases, Ho Chi Minh City, Vietnam and by the Oxford University Tropical Research Ethics Committee (OxTREC), Oxford, UK. Written informed consent was obtained from parents or legal guardians of children prior to enrolment into the study.

### Diagnostic testing

RNA extraction from nasopharyngeal swabs was done as described previously [11]. RNA was analyzed by multiplex real-time RT-PCR on a Roche Light Cycler 480 II Thermocycler (Roche Diagnostics, Penzberg, Germany) [9, 10] and RSV Locked Nucleic Acid (LNA) Real-time RT-PCR (LNA assay) on a DNA Engine Peltier Thermocycler and Chromo 4 Real-time PCR system detector (Bio-Rad, Hercules (CA), USA) [11]. The multiplex real-time PCR detects 13 other human respiratory viruses: influenza virus A (flu A); influenza virus B (flu B); adenovirus (AdV), enterovirus (EnV), human metapneumovirus (MPV), human coronaviruses (CoV-229E, OC43, HKU1, SARS CoV & NL63), human rhinovirus (RV A, B and C), parainfluenza virus 1, 2 and 3, 4 (PIV1, 2, 3, 4), parechovirus (PeV) and human bocavirus (BoV) [9, 10]. The LNA assay assesses viral load (log10 copies/ml) of RSV subgroups A and B (RSV A, RSV B) [11]. For every RSV positive patient, the second sample (at discharge or day 7) was also assessed by LNA assay.

### Data analysis

#### Definitions

Severe disease on admission was defined as having an oxygen saturation of SpO2<92 or requiring supplemental oxygen or ventilatory support (by nasal cannula, nasal continuous positive airway pressure (NCPAP), mask CPAP or mechanical ventilation/intubation) or clinical cyanosis. A clinical severity score (adapted from [12–15]) was introduced to grade the clinical status of patients at enrolment (Table 1). Only patients from whom all components in the score table were available were given a clinical severity score. Long hospitalization was defined as longer than 7 days.

**Table 1:** Clinical score index*.

Clinical symptoms	Score given
Mild fever (37.5 to 38.5°C)	1
High fever (>38.5°C)	2
Respiratory rates from 45–59 per min	1
Respiratory rates from 60–74 per min	2
Respiratory rates from > 75 per min	3
Chest indrawing	2
Wheezing	1
Stridor	1
Apnea	3
Cyanosis	3
Low SpO_2_ (<92) or under oxygen	3
NCPAP or CPAP or ventilated during hospitalization	4
High ALT**	1
High AST**	1

* A higher clinical score indicates more severe disease.

** Liver enzymes ALT and AST levels were considered to be elevated if above 36 IU/l and above 58 IU/l, respectively.

#### Statistical analysis

Associations between categorical and continuous variables were analyzed using the Mann-Whitney-U test or Kruskal-Wallis test for continuous variables, and the Fisher exact test for dichotomous variables. Spearman’s rank correlation coefficient was used to assess a general monotonic trend between two continuous variables. A simple linear regression model was used to measure linear associations between RSV viral load and age or day of illness or number of leucocytes in blood. Multivariable logistic regression analysis was performed to assess risk factors for severe disease or long hospitalization. The following variables were considered for the models: age, sex, premature birth, previous hospitalization for respiratory illness, other household members sick at home, living with smoker(s), number of members at home, fever, RSV infection (RSV positivity, viral load, and subgroup) and RV infection (RV positivity as single infection or co-infection with RSV). The models’ predictive ability was investigated by calculating the area under the receiver operating characteristic (ROC) curve of the model (AUC), i.e the higher the AUC the better prediction power the model has. The Hosmer—Lemeshow goodness-of-fit test was done to assess model adequacy. The Hosmer-Lemeshow test of goodness-of-fit tests the null hypothesis that there is no difference between the observed and predicted values of the response variables. Therefore, when the test is not significant (p>0.05) the null hypothesis cannot be rejected and this means that the model fits the data well. Risk factors for disease severity or long hospitalization were further assessed using odds ratios (OR) and 95% confidence intervals (95% CIs). All statistical tests were performed as two-tailed tests at 5% significance in either R version 2.13.1 (R Foundation for Statistical Computing, Vienna, Austria) or Intercooled Stata version 9.2 (College Station, TX, USA).

## Results

### Demographic, clinical features and viral etiologies of study population

**Fig 1:**
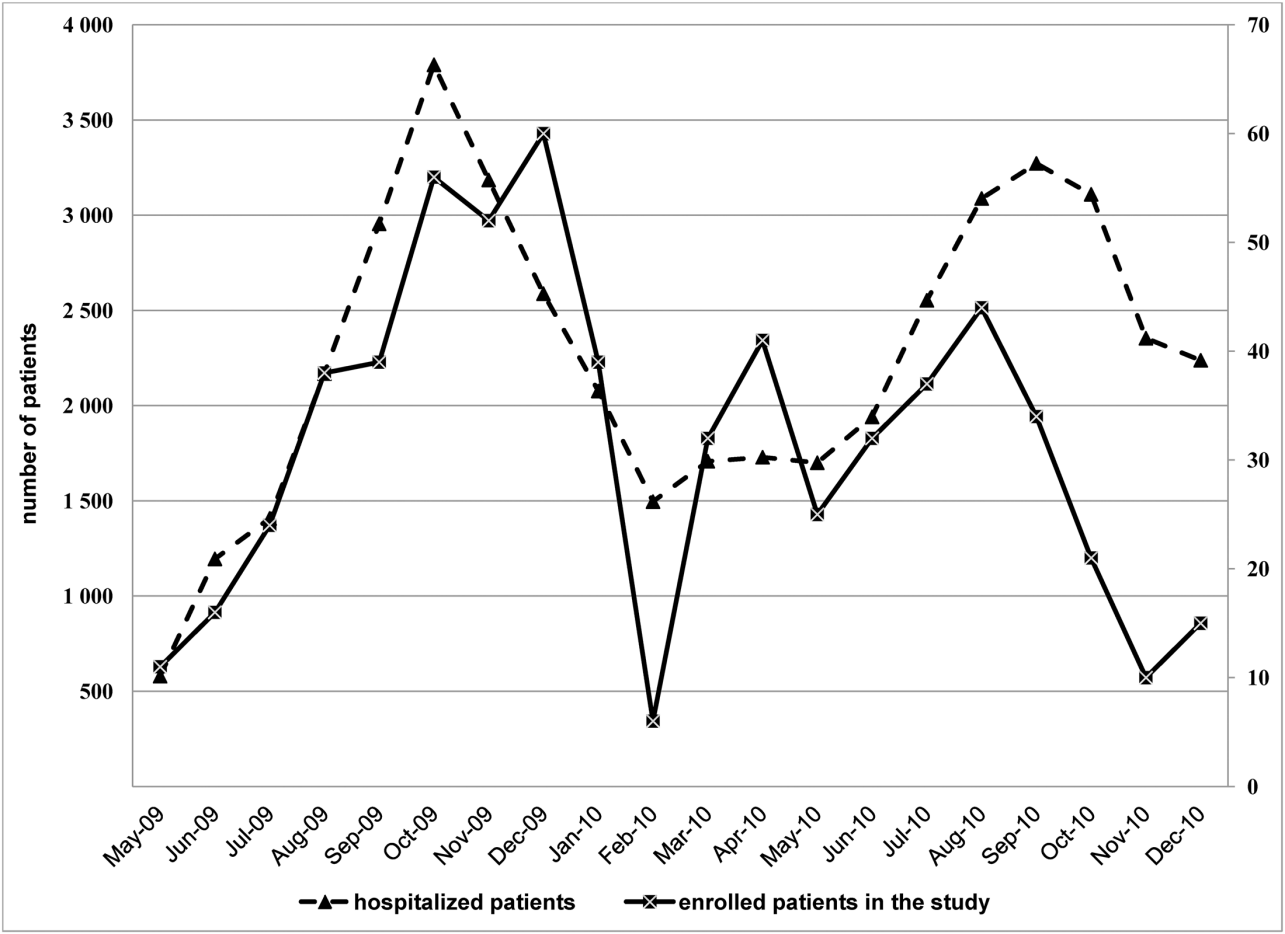
Number of cases enrolled and total numbers of LRTI children hospitalized in two referral pediatric hospitals in southern Vietnam, May 2009 to December 2010. A total of 106/632 (17%)of cases met at least one clinical criterion for severe disease, and 43% (271/632) were hospitalized for >7 days. Among the children requiring supplemental oxygen or ventilatory support, 16/93 (17%) received NCPAP, 2/93 (2%) were intubated.

A total of 632 children aged 1–24 months (median 7, IQR 4–12 months) were enrolled into the study between May 2009 and December 2010. 10/632 (2%) patients were admitted to ICU, 36/632 (6%) to the ECU and 586/632 (92%) to the Respiratory Ward. The monthly distribution of children hospitalized for LRTI and enrolled in this study during the study period are shown in Fig 1.

Diagnoses on admission were based on clinical symptoms, routine laboratory tests and chest radiography; physicians were unaware of viral diagnostic laboratory results. 438/632 (69%) patients were diagnosed with bronchiolitis; 164/632 (26%) with pneumonia, 22/632 (3%) with combined bronchiolitis and pneumonia, 3/632 (0.4%) with laryngotracheitis, and 5/632 (0.8%) with undifferentiated LRTI. In addition to respiratory symptoms, 21/632 (3%) patients had diarrhoea on admission, and 4/632(0.6%) had congenital heart disease (ventricular or atrial septum defects). Only 18/598 (3%) had blood culture test which was part of standard clinical care at hospitals and only 5/18 were positive (*1 Staphylococcus aureus*, *1 Streptococcus pneumoniae*, 1 *Haemophilus influenzae*, 1 *Pseudomonas aeruginosa*, 1 *Klebsiella spp)*. 535/632 (85%) children received antibiotics, 100% children with clinical diagnosis of pneumonia received antibiotics.

Discharge information was available for 596/632 (94%) cases: 363/596 (61%) of patients fully recovered; 226/596 (38%) had incomplete recovery at the time of discharge; 4/596 (1%) went home without permission (mostly due to economic reasons, patients could not pay hospitalization fee) or formal hospital discharge; and 3 patients (1%) died in the hospital. One fatal case (8 months old) was diagnosed with septicemia and severe pneumonia (*S*. *pneumoniae* was recovered from blood culture; no respiratory viruses were detected in nasopharyngeal swabs) and died on the second day of admission. The two other fatal cases were 6 and 3 months old and did not have any severe indications on admission but deteriorated quickly after 5 days of hospitalization. No organisms were recovered from blood culture and virology results yielded a single RV infection and a triple infection (RV, PIV-3 and BoV), respectively.

Viral etiologies were identified in the vast majority (91%, 574/632) of patients; single viral infections accounted for 375/632 (59%) of cases and co-infections with multiple viruses were found in 199/632 (31%) (Table 2). RSV was the most frequently detected: overall (302/632, 48%) and in single infections, while RV was most frequently detected in co-infections (Table 2).

**Table 2:** Viral single and co-infections among all enrolled patients.

VIRUSES	All	Single infections	Co- infection	p-value
	N = 632	N = 375	N = 199	
RSV, n(%)	302 (48)	201 (54)	101 (51)	0.9^(2)^
RSV A, n(%)	156 (25)	101 (27)	55 (28)	0.9^(2)^
Median log copies/ml RSVA (IQR)	7.5 (6.7–8.2)	7.7 (6.7–8.1)	7.4 (6.3–8.2)	0.2^(1)^
RSV B, n(%)	139 (22)	93 (25)	46 (23)	0.7^(2)^
Median log copies/ml RSVB (IQR)	7.6 (6.9–8.2)	7.7 (7.1–8.2)	7.5 (6.6–8.0)	0.2^(1)^
Flu, n(%)	16 (3)	8 (2)	8 (4)	0.2^(2)^
Flu A, n(%)	10 (2)	5 (1)	5 (3)	0.3^(2)^
Median Cp-value flu A (IQR)	31 (29–34)	30 (30–31)	33 (29–35)	0.5^(1)^
InflV B, n(%)	6 (1)	3 (1)	3 (2)	0.4^(2)^
Median Cp-value flu B (IQR)	31 (29–33)	32 (29–33)	30 (20–34)	0.8^(1)^
AdV, n(%)	37(6)	3 (1)	34 (17)	**0.001**^(2)^
Median Cp-value AdV (IQR)	35 (31–36)	35 (30–35)	34 (31–36)	0.8^(1)^
EnV, n(%)	65 (10)	2 (1)	63 (32)	**0.001**^(2)^
Median Cp-value EnV (IQR)	33 (30–35)	32 (29–35)	33 (30–35)	0.8^(1)^
MPV, n(%)	30 (5)	17 (5)	13 (7)	0.3^(2)^
Median Cp-value MPV (IQR)	27 (24–36)	26 (23–29)	36 (27–36)	**0.02**^(1)^
RV, n(%)	206 (33)	87 (23)	119 (60)	**0.001**^(2)^
Median Cp-value RV (IQR)	27 (25–30)	26 (24–28)	28 (26–31)	**0.001**^(1)^
PIV-1, n(%)	9 (1)	5 (1)	4 (2)	0.5^(2)^
Median Cp-value PIV-1 (IQR)	27 (25–30)	28 (26–30)	26 (22–32)	0.5
PIV-2, n(%)	14 (2)	4 (1)	10 (5)	**0.007**^(2)^
Median Cp-value PIV-2 (IQR)	26 (23–30)	22 (22–23)	33 (26–36)	**0.03**^(1)^
PIV-3, n(%)	47 (7)	18 (5)	29 (15)	**0.001**^(2)^
Median Cp-value PIV-3 (IQR)	29 (24–35)	25 (23–32)	32 (25–37)	**0.03**^(1)^
PIV-4, n(%)	25 (4)	13 (4)	12 (6)	0.2^(2)^
Median Cp-value PIV-4 (IQR)	31 (27–34)	29 (25–33)	33 (30–35)	**0.04**^(1)^
hCoV, n(%)	25 (4)	8 (2)	17 (9)	**0.001**^(2)^
Median Cp-value hCoV (IQR)	29 (27–32)	27 (24–29)	30 (28–33)	0.1^(1)^
PeV, n(%)	12 (2)	2 (1)	10 (5)	**0.001**^(2)^
Median Cp-value PeV (IQR)	28 (26–29)	28 (26–31)	28 (27–29)	0.7^(1)^
BoV, n(%)	42 (7)	7 (2)	35 (18)	**0.001**^(2)^
Median Cp-value BoV (IQR)	29 (25–33)	23 (22–30)	30 (25–35)	0.1^(1)^

P-value was calculated between single infections and co-infections groups,

^(1)^ Mann-Whitney test,

^(2)^ Fisher’s exact test,

N.A: not applicable.

A significantly higher proportion of RV, EnV, BoV, AdV, PIV-2, PIV-3, CoV and PeV were detected among co-infections when compared to the single infections (Table 2). For MPV, RV, PIV-2, PIV-3 and PIV-4, the relative viral load in single infections was significantly higher than in co-infections (Table 2). RSV-RV co-infection was the most common double infection, identified in 147/632 (23%) cases. Triple infections were identified in 47/632 (7%) and in 5/632 cases, 4 different viruses were detected (RSV-RV-PIV3-CoV, EnV-RV-CoV-BoV, AdV-EnV-RV-PIV2; RSV-AdV-EnV-RV).

### Risk factors related to severe LRTI cases

In the univariate analysis, LRTI severity was associated with younger age (median age in months [IQR] = 5 [3–10] in severe cases versus 7 [4–12] in non-severe cases, Mann- Whitney test p-value = 0.001 –S1 Fig), presence of fever (72%, 76/106 in severe cases versus 47%, 247/526 in non-severe cases, Fisher exact p-value = 0.001), living in a household with a high number of people (median household members [IQR] = 5 [4–6] in severe cases versus 4 [3–5] in non- severe cases, Mann-Whitney test p-value = 0.03), or having other household members sick at home (50%, 52/103 in severe cases versus 37%, 193/528 in non-severe cases, Fisher exact p-value = 0.001). Co-infections were more frequent among the non-severe cases, and the clinical severity score was inversely correlated to the number of viruses detected (Spearman cor = -0.09, p = 0.04). Among the single infections, viral load was not associated with severity. For RV, single infections were significantly more common (21%, 22/106) in severe cases versus in non-severe cases (12%, 65/526) (Fisher’s exact test P-value = 0.02). The relationship between RV and severity was also observed in increased odds ratios for elevated (worse) clinical score (OR = 1.86, 95%CI (1.08–3.17), p = 0.02). Similar relationships were not observed for RSV single infection cases.

In multivariate analyses, significant predictors of severity within the entire study population were younger age, presence of fever, and RV single infection (Table 3). The Hosmer—Lemeshow goodness-of-fit test result was χ^2^ = 5.94, p-value = 0.65) and the AUC for the prediction model was 0.74. Similarly, predictors of long hospitalization were younger age, presence of fever, and previous hospitalization with respiratory illness were significant (Table 3). The Hosmer—Lemeshow goodness-of-fit test result was χ^2^ = 7.38, p-value = 0.5) and the area under the ROC curve for the prediction model of long hospitalization was 0.66. RSV infection was not correlated to disease severity, or to duration of hospitalization (Table 3).

**Table 3:** Factors independently predicting disease severity or long hospitalization among LRTI hospitalized children and among RSV-infected children.

Independent predictors	Outcome measure							
	Disease severity				Long hospitalization			
	LRTI children (N = 605)		RSV-infected children (N = 293)		LRTI children (N = 605)		RSV-infected children (N = 293)	
	OR(95%)	P-value	OR(95%)	P-value	OR(95%)	P-value	OR(95%)	P-value
**Age (months)**	0.89 (0.85–0.94)	**0.001**	0.89 (0.81–0.95)	**0.002**	0.93 (0.90–0.96)	**0.001**	0.95 (0.91–1.00)	**0.065**
**Male gender**	0.83 (0.51–1.36)	0.46	0.92 (0.44–1.90)	0.83	1.17 (0.81–1.69)	0.39	1.41 (0.82–2.43)	0.21
**Premature birth**	1.06 (0.47–2.39)	0.88	1.20 (0.37–3.89)	0.75	1.79 (0.96–3.29)	0.06	2.34 (0.91–5.99)	0.07
**Previous hospitalization with respiratory illness**	0.99 (0.53–1.83)	0.96	0.43 (0.13–1.42)	0.17	2.49 (1.59–3.92)	**0.001**	1.29 (0.61–2.72)	0.49
**Other household members sick at home**	1.41 (0.87–2.26)	0.15	1.98 (0.99–3.94)	**0.05**	1.15 (0.81–1.64)	0.42	1.26 (0.74–2.14)	0.38
**Having smokers at home**	1.31 (0.81–2.11)	0.26	1.24 (0.63–2.45)	0.53	0.92 (0.66–1.31)	0.67	1.31 (0.79–2.17)	0.28
**Number of members at home**	1.04 (0.95–1.15)	0.88	1.03 (0.89–1.10)	0.63	1.02 (0.97–1.11)	0.49	0.95 (0.85–1.07)	0.42
**Fever**	3.82 (2.29–6.36)	**0.001**	4.84 (2.27–10.30)	**0.001**	1.77 (1.25–2.51)	**0.001**	2.66 (1.58–4.47)	**0.001**
**RSV infection**^(1)^	1.39 (0.82–2.39)	0.22	0.60 (0.30–1.17)	0.13	0.79 (0.54–1.15)	0.22	0.79 (0.48–1.30)	0.36
**RV infection**^(2)^	2.31 (1.17–4.59)	**0.02**	0.30 (0.08–1.07)	0.06	1.1 (0.63–1.84)	0.77	1.17 (0.58–2.35)	0.65
**RSV viral load (log copies/ml)**	N.A	N.A	1.03 (0.78–1.35)	0.85	N.A	N.A	1.14 (0.93–1.39)	0.19

^(1)^ RSV single infection factor was used for the model of the whole population and RSV subgroup (RSV B versus A) was used for the model of RSV-infected patients

^(2)^ RV single infection factor for the model of the whole population and RV co-infection with RSV for the model of RSV-infected patients

N.A: not applicable

### Demographic, clinical characteristics and seasonal variations of RSV infections versus other viral infections

RSV infections occurred at a younger age than the 3 other leading single viral infections, i.e RV, MPV, PIV-3 (Tables 4 and 5), and were significantly more frequent in the first 6 months of life (Fisher exact test p-value = 0.001). In contrast, none of the other viruses exhibited significant age group distributions (Tables 4 and 5). A significant inverse correlation was observed between RSV load at enrolment and patient age. Indeed, on average RSV viral loads decreased by 0.03 log copies/ml per month increase in age (t = -2.37, p-value = 0.02).

**Table 4:** Viral etiologies and age group.

Virus name	Age groups				
	<2m	2m-6m	7m-11m	12m–24m	Total
	n = 41	n = 277	n = 174	n = 140	N = 632
RSV positive, n(%)	22 (54)	152 (55)	74 (43)	54 (39)	302 (48)
RSVA, n(%)	13 (32)	84 (30)	31 (18)	28 (20)	156 (25)
RSV B, n(%)	9 (22)	63 (23)	42 (27)	25 (18)	139 (22)
Both RSVA and RSVB, n(%)	0 (0)	5 (2)	1 (1)	1 (1)	7 (1)
Flu V positive, n(%)	0 (0)	6 (2)	5 (3)	5 (4)	16 (3)
Flu A, n(%)	0 (0)	4 (1)	4 (2)	2 (1)	10 (2)
Flu B, n(%)	0 (0)	2 (1)	1 (1)	3 (2)	6 (1)
AdV, n(%)	1 (2)	11 (4)	15 (9)	10 (7)	37 (6)
EnV, n(%)	5 (12)	29 (10)	17 (10)	14 (10)	65 (10)
MPV, n(%)	3 (7)	9 (3)	9 (5)	9 (6)	30 (5)
RV, n(%)	10 (24)	91 (33)	61(35)	44 (31)	206 (33)
PIV-1, n(%)	0 (0)	5 (2)	2 (1)	2 (1)	9 (1)
PIV-2, n(%)	0 (0)	8 (3)	5 (3)	1 (1)	14 (2)
PIV-3, n(%)	2 (5)	16 (6)	14 (8)	15 (11)	47 (7)
PIV-4, n(%)	0 (0)	12 (4)	7 (4)	6 (4)	25 (4)
CoV, n(%)	1 (2)	14 (5)	6 (3)	4 (3)	25 (4)
PeV, n(%)	0 (0)	9 (3)	2 (1)	1 (1)	12 (2)
BoV, n(%)	3 (7)	13 (5)	12 (7)	14 (10)	42 (7)
**Single infections**, n(%)	26 (63)	160 (58)	104 (60)	85 (61)	375 (59)
**Co-infections**, n(%)	9 (22)	94 (34)	55 (31)	41 (29)	199 (31)
**Negative cases**, n(%)	6 (15)	23 (8)	15 (9)	14 (10)	58 (9)

**Table 5:** Comparison of demographic and clinical characteristics among patients with RSV, RV, MPV and PIV-3.

	Single RSV infection	Single RV infection		Single MPV infection		Single PIV-3 infections	
	N = 201	N = 87	p-value	N = 17	p-value	N = 18	p-value
**DEMOGRAPHIC**							
Median age in months (IQR)	6 (3–10)	8 (4–12)	0.09^(1)^	11 (8–14)	**0.02**^(1)^	9 (6–16)	**0.05**^(1)^
Infant (less than 2months), n(%)	15 (7)	5 (6)		2 (12)		2 (11)	
Infant (2 to 6months), n(%)	103 (51)	31 (36)		2 (12)		3 (17)	
Infant (7 to 11months), n(%)	47 (24)	31 (36)		6 (35)		7 (39)	
Infant (12 to 24months), n(%)	36 (18)	20 (23)		7 (41)		6 (33)	
Male, n(%)	138 (69)	62 (71)	0.7^(2)^	12 (71)	0.9^(2)^	10 (56)	0.3^(2)^
Median number of household members (IQR)	4 (4–6)	4 (4–6)	0.7^(1)^	4 (3–5)	0.3^(1)^	4 (3–6)	0.4^(1)^
Living with smokers, n (%)	102/196 (52)	51/86 (59)	0.3^(2)^	5 (29)	0.07^(2)^	12 (67)	0.2^(2)^
**MEDICAL STORY**							
Median birth weight (kg) (IQR)	3.9 (2.2–3.3)	2.7 (2.3–3.2)	0.3^(1)^	3.3 (2.8–3.8)	0.5^(1)^	3 (2.6–3.4)	0.7^(1)^
Breastfeeding, n(%)	148/197 (75)	59/86 (69)	0.3^(2)^	16 (94)	0.07^(2)^	13 (72)	0.8^(2)^
Premature birth, n(%)	20/199 (10)	14/85 (16)	0.1^(2)^	0/16 (0)	N.A	1/17 (6)	0.5^(2)^
Daycare, n (%)	25/197 (13)	10/84 (12)	0.9^(2)^	2/16 (13)	1.0^(2)^	5/18 (28)	0.08^(2)^
Previous hospitalization with respiratory illness, n (%)	22 (11)	23 (26)	**0.001**^(2)^	2 (12)	0.9^(2)^	3 (17)	0.5^(2)^
Other household members sick at home, n (%)	76/197 (39)	36/85 (42)	0.6^(2)^	5 (29)	0.5^(2)^	5 (28)	0.4^(2)^
**CLINICAL CHARACTERISTICS**							
Fast breathing, n(%)	166 (83)	75 (86)	0.4^(2)^	14 (82)	1.0^(2)^	14 (78)	0.6^(2)^
Cyanosis, n(%)	6 (3)	3 (4)	N.A	0 (0)	N.A	0 (0)	N.A
Chest indrawings, n(%)	193 (96)	81 (94)	0.3^(2)^	15 (88)	0.1^(2)^	15 (83)	0.01^(2)^
Stridor, n(%)	1 (1)	1 (1)	0.5^(2)^	0 (0)	N.A	0 (0)	N.A
Wheezing, n(%)	192 (96)	76 (88)	**0.01**^(2)^	16 (94)	0.8^(2)^	17 (94)	0.8^(2)^
Fever (>37.5°C), n(%)	112 (56)	50 (57)	0.8^(2)^	7 (41)	0.2^(2)^	6 (33)	0.07^(2)^
Fever (≥ 38.5°C), n(%)	46 (23)	13 (15)	0.1^(2)^	5 (29)	0.5^(2)^	2 (11)	0.2^(2)^
Rash, n(%)	2 (1)	3 (3)	0.1^(2)^	0 (0)	N.A	1 (6)	0.1^(2)^
Runny nose, n(%)	181 (91)	70 (81)	**0.03**^(2)^	15 (88)	0.8^(2)^	17 (94)	0.5^(2)^
Low SpO_2_, n(%)	19/140 (14)	6/60 (10)	0.5^(2)^	1/14 (7)	0.5^(2)^	1/17 (6)	0.4^(2)^
Median of duration of hospitalization (IQR)	6 (5–7)	6 (5–8)	**0.02**^(1)^	6 (4–7)	0.9^(1)^	6 (5–7)	0.9^(1)^
Duration of hospitalization ≥ 7 days, n(%)	77 (38)	43 (49)	0.08^(2)^	7 (41)	0.8^(2)^	7 (39)	1.0^(2)^
High ALT, n(%)	54 (27)	16 (18)	0.1^(2)^	2 (12)	0.2^(2)^	3 (17)	0.3^(2)^
High AST, n(%)	44 (22)	9 (10)	**0.02**^(2)^	4 (23)	0.9^(2)^	3 (17)	0.6^(2)^
Median of number of white cells in blood (K/mm^3^) (IQR)	10 (8–14)	13 (11–17)	**0.001**^(1)^	11 (9–13)	0.8^(1)^	11 (8–17)	0.6^(1)^
Severe cases, n(%)	36 (18)	22 (25)	0.2^(2)^	3 (18)	1.0^(2)^	3 (17)	1.0^(2)^
Median of clinical score (IQR)	6 (4–7) (n = 186)	5 (4–7) (n = 80)	0.5^(1)^	5 (4–6)	0.4^(1)^	4.5 (4–6)	**0.02**^(1)^
**DIAGNOSIS**							
Bronchiolitis, n(%)	162 (81)	65 (75)	0.3^(2)^	10 (59)	0.06^(2)^	9 (50)	0.005^(2)^
**TREATMENTS**							
Antimicrobial agents, n(%)	169 (84)	62 (71)	**0.01**^(2)^	17 (100)	0.07^(2)^	17 (94)	0.2^(2)^
Corticosteroids, n(%)	9 (4)	6 (7)	0.4^(2)^	1 (6)	0.8^(2)^	0 (0)	N.A
Bronchodilators, n(%)	168 (84)	67 (77)	0.2^(2)^	9 (52)	**0.002**^(2)^	10 (56)	**0.004**^(2)^
Supplemental oxygen, n(%)	31 (15)	21 (24)	0.08^(2)^	2 (12)	0.7^(2)^	2 (11)	0.6^(2)^
**OUTCOMES**							
Full recovery, n(%)	119/195 (61)	46/81 (57)	0.5^(2)^	11 (73)	0.8^(2)^	11 (65)	0.9^(2)^
Severe cases, n(%)	36 (18)	22 (25)	0.2^(2)^	3 (18)	1.0^(2)^	3 (17)	0.9^(2)^
Death, n(%)	0 (0)	1(1)	N.A	0 (0)	N.A	0 (0)	N.A

p-values were based on comparison with children having RSV single infections.

^(1)^ Mann-Withney test,

^(2)^ Fisher’s exact test,

N.A: not applicable

A higher clinical severity score was found only among RSV single infection cases compared to those of PIV-3 single infection cases (Mann-Withney test p-value = 0.02, Table 5), but not in any of the other pair-wise comparisons. While RSV single infection had a significantly shorter duration of hospitalization, a higher prevalence of elevated AST and a lower leucocyte count than RV single infection cases (Table 5, Mann-Withney test p-value = 0.02, 0.02 and 0.001, respectively). We also observed a significantly lower number of leucocytes in RSV single infection versus those in RSV co-infection (Mann-Withney p-value = 0.001), and an inverse correlation between RSV load in nasopharyngeal swabs and leucocyte count in blood collected at enrolment (coeff = -0.56, 95%CI = -0.98 to -0.14, p-value = 0.009).

Among 302 RSV positive cases, 156/302 (52%) were RSV A, 139/302 (46%) were RSV B, and 7/302 (2%) were both RSV A and B. There was no difference in proportion of A and B subgroups between single and co-infections, and no difference in viral load among subgroups at enrolment (Table 2).

**Fig 2:**
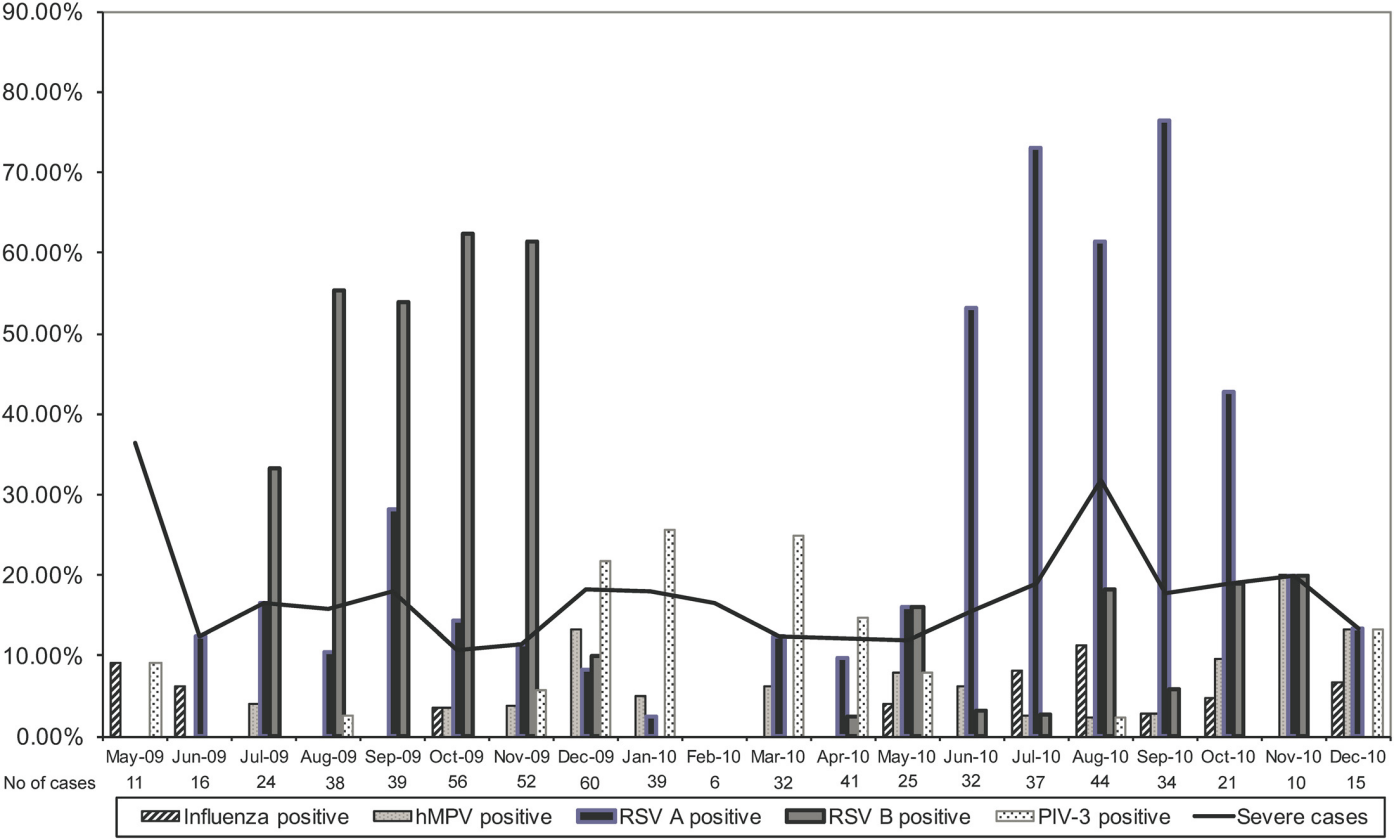
RSV, RSV subgroups, other viruses and severe cases distribution.

Strong seasonal variations of RSV prevalence with peaks during the rainy season from May to October were observed over the two seasons of the study. RSV B was dominant during the first season and RSV A during the second season (Fig 2). Overall case numbers for MPV and influenza were insufficient to detect patterns of seasonality.

### Risk factors related to severe RSV-infected LRTI cases

The most frequently detected viruses in RSV co-infection were RV, EnV and AdV (S1 Table). In the univariate analysis, the number of viruses co-detected with RSV or the RSV viral load was not associated with RSV severity, or with clinical severity score (cor = 0.06, p = 0.1 and cor = -0.3, p = 0.2, respectively). RSV patients with long duration of hospitalization had a significantly higher RSV viral load than others (median of RSV load (IQR) 8 (7–8) versus 7 (7–8) Mann-Withney test p-value = 0.004, respectively). No associations with RSV subgroups and severity or duration of hospitalization were found.

In multivariate analyses for the RSV-infected population, the logistic regression analysis of ten selected predictors identified age (OR = 0.88, 95%CI:0.81–0.95, p-value = 0.001), fever (OR = 4.80, 95%CI:2.26–10.19, p-value = 0.001), and having another household member sick at home (OR = 2.04, 95%CI: 1.03–4.02, p-value = 0.04) as independent variables associated with disease severity in RSV-infected children (Table 3). The Hosmer—Lemeshow goodness-of-fit test result was χ^2^ = 5.73, p-value = 0.68) and the area under the ROC curve for the prediction model was 0.76. For risk factors of long hospitalization among RSV patients, only fever (OR = 2.67, 95%CI: 1.59–4.48, p-value = 0.001) was a significant independent predictor (Table 3). The Hosmer—Lemeshow goodness-of-fit test result was χ^2^ = 9.35, p-value = 0.31 and the AUC for the prediction model of long duration hospitalization was 0.67. Again, RSV subgroups or RSV viral load were not associated with disease severity or long hospitalization in multivariate analyses.

## Discussion

Over the last decade, multiplex molecular diagnostics have revolutionized the diagnostics of respiratory infections and greatly expanded the available data on viral etiologies and coinfection [6, 16–22]. Here, using a previously described multiplex assay to detect 13 different respiratory viruses, viruses were identified in 91% of enrolled patients, and viral co-infection was found in 31%. The high prevalence of viral infections among LRTI cases, the predominance of RSV and RV infections, and the co-infection rates between RSV and RV, EnV and AdV in our study were similar to other published work in comparable populations [21, 23]. In these previous studies, among hospitalized children, the proportion of viral causes ranged from 93–97%; RSV and RV were the leading causes, ranging from 64 to 73% and from 30–34%, respectively [21, 23].

Our study confirms the importance of RSV infection in children under two as shown by many other studies [17, 21, 23–25] and is the first study to examine the demographic, clinical and virological characteristics of RSV infections in South Vietnam. Our results confirm and extend previous observations regarding associations of RSV infections with young age compared to the 3 other leading viral infections (rhinovirus (RV), metapneumovirus, parainfluenza virus (PIV-3), wheezing, runny nose, leucopenia (among RSV single versus RSV co-infection or RV single infection) and risk factors (premature birth) [17, 21, 23–26]. In addition, we observed a significant negative correlation between RSV load in nasopharyngeal swabs and leucocyte count in blood collected at enrolment (coeff = -0.56, 95%CI = -0.98 to -0.14, p-value = 0.009). These findings are consistent with previous reports [26, 27] and it could be hypothesized that leukocytes in RSV infection are being recruited at specific sites away from the circulating blood [28, 29].

One of our aims was to determine risk factors for severity and long hospitalization among all study patients and among RSV-infected patients. We observed that RSV load at enrolment was significantly related to long hospitalization in univariate analysis, but this was not confirmed in the multivariate analysis; and RSV infection, RSV viral load, RSV subgroups and RSV slope (i.e RSV load dynamic between two time points: admission and discharge, it was calculated by dividing the difference of viral load at admission and at discharge by the number of days between these two time points—data not shown), did not correlate to disease severity or long hospitalization. Only young age and fever were independent predictors for disease severity in both populations (study population and RSV population). Reports about the relation between RSV infection [30, 31] or RSV load [12, 26, 32, 33] or RSV subgroups [34–38] and disease severity have been contradicting. Marguet *et al*. reported that RSV infection caused more severe disease than RV infection among children under 1 year of age. In a study by Tran *et al*. conducted in Children Hospital 2, Ho Chi Minh City, targeting hospitalized children under 15 years, RSV positive children had significantly higher clinical severity scores compared to RSV-negatives. However, this comparison was based on univariate analysis without considering other confounding factors such as age [6]. Papadopoulos *et al*. found, among children less than 18 months, that the presence of RV increases the risk for severe disease significantly, by approximately five-fold. RSV infection was also correlated with severity, however, this did not reach significance [30, 31]. RSV loads at the second collection had a significant negative correlation (correlation = -0.33, p-value = 0.009) with day of illness, while this correlation was not found for the enrolment samples which were collected on any day during the first days of illness (S2 Fig). This RSV load trend during RSV infection was also observed in other studies [39] and confirmed the study samples were collected on the early day of RSV illness.

Thus far, observations regarding the relation between viral co-infections and disease severity have been contradictory and biased by different study designs and viral diagnostic tools used [24, 40]. Similar to other studies, we observed a significantly lower proportion of co-infections in severe cases compared to non-severe cases [21, 41]. However, among RSV infected cases, we did not observe significant differences in disease severity between single and co-infected cases. In addition, there were significant differences between RSV-RV coinfections (3/45, 6.6%) versus RSV-EnV (9/27, 75%)–Fisher’s exact p-value = 0.006 but no significant versus RSV-AdV co-infections (2/18, 11%). Interestingly, and in agreement with Papadopoulos *et al*. [31], although RV was detected mostly as a coinfection, RV single infection was identified as an independent risk factor of severe disease. This finding should be interpreted with caution because the role of RV in the pathogenesis of severe LRTI remains a topic of debate [30, 31, 42–47]. Cross-reactivity in the PCR detection of EnV and RV using 5’UTR primers is a known problem, particularly for EV-D68 which has a rhinoviral 5’ UTR sequence, and this complicates ascertainment of EnV and RV diagnoses [48].

Despite the fact that the study population from our current study and our previous study [3] and from other studies [2, 6] consisted of hospitalized children and is therefore not representative of Vietnamese children in general, a consistent RSV seasonal pattern with peaks during the rainy season (between May and October) was observed from 2005 to 2010. The clear shift of RSV subgroups was not observed during the period 2005 to 2007 [3] but we observed this in our current study and Tran et al. also confirmed the dominance of RSV A during the same period [6]. In many studies, RSV has been reported as a highly seasonal infection and RSV outbreaks are frequently associated with the rainy season in areas with tropical and sub-tropical climate [5, 49]. Possible explanations for this include meteorological effects such as humidity and UVB radiation on the environmental stability of RSV viruses. The stability of RSV in aerosols was shown to increase with higher humidity [50]. Moreover, population behaviors as staying indoors during cold or rainy seasons may facilitate transmission of RSV.

From this cohort, we have recently reported analysis of RSV whole genomes [51] and are currently analysing host expression profiles of blood and nasopharyngeal swabs at two time points (manuscript in preparation).

There are a number of limitations to our study. Firstly, few bacterial diagnostic results were obtained from patients. The role of bacteria as a cause of LRTI or as cause of superinfection, especially in RSV and influenza virus infection [52], is important for antibiotic intervention strategies. Lower airway secretions (sputum for example) are considered the optimal specimen type for detecting bacterial (co-) infection, yet are often difficult to obtain from young children, and can only be readily obtained from intubated children. Secondly, only a limited number of patients were enrolled as compared to the total number of LRTI hospitalizations in two hospitals during the study period (632/45134, 1%) although the number of enrolled patients followed a similar pattern as the total number of patients (Fig 1).

In summary, our study has contributed detailed clinical and virological data on RSV and other viruses in respiratory infections among children under 2 years old, the most vulnerable age group, in a lower middle income setting in Asia: Vietnam. The data on seasonality of viruses are crucial for health care management, such as preparedness for the annual epidemics in terms of hospital capacity and RSV prevention in high-risk children using Palivizumab.

## Supporting Information

S1 Fig Age and severity. (TIF)

S2 Fig RSV viral load and day of illness. **(A)** The first 4 days of illness. (B) Day of follow-up (after day 4 of illness).
(TIF)

S1 Table Viral etiologies identified among RSV co-infection cases. (DOCX)
