# Supplementary figures and images for: Respiratory Syncytial Virus and Other Viral Infections among Children under Two Years Old in Southern Vietnam 2009-2010: Clinical Characteristics and Disease Severity
Source: PLoS One. 2016 Aug 8;11(8):e0160606. doi: 10.1371/journal.pone.0160606 (PMC4976934; doi:10.1371/journal.pone.0160606)

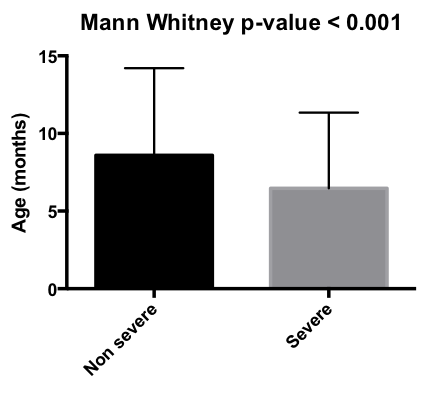

Supplement: S1 Fig — (TIF) [file pone.0160606.s001.tif]

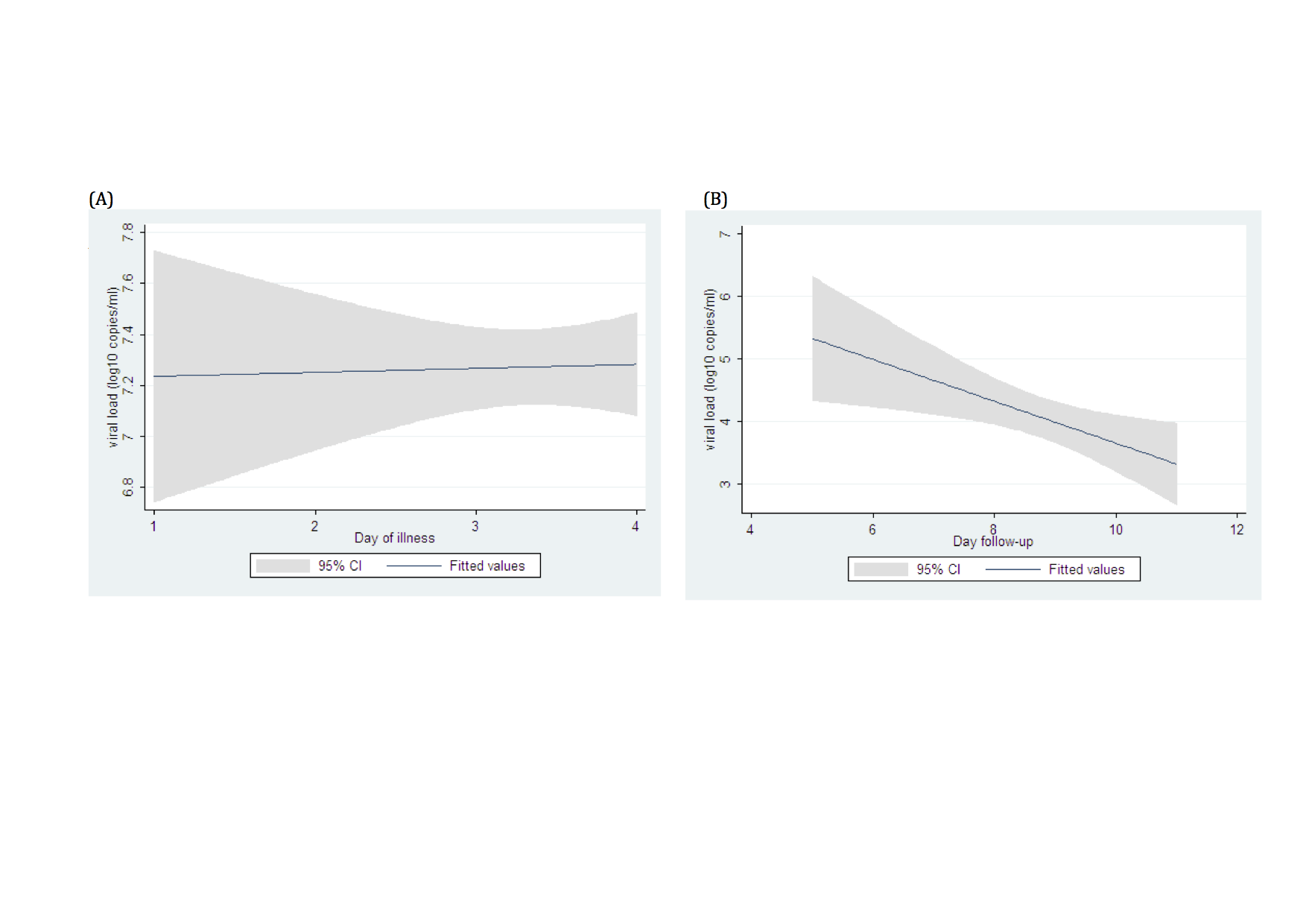

Supplement: S2 Fig — (A) The first 4 days of illness. (B) Day of follow-up (after day 4 of illness). (TIF) [file pone.0160606.s002.tif]
