# Supplementary material for: Respiratory Syncytial Virus and Other Viral Infections among Children under Two Years Old in Southern Vietnam 2009-2010: Clinical Characteristics and Disease Severity
Source: PLoS One. 2016 Aug 8;11(8):e0160606. doi: 10.1371/journal.pone.0160606 (PMC4976934; doi:10.1371/journal.pone.0160606)
Supplement: S1 Table — (DOCX) [file pone.0160606.s003.docx]

**S1 Table. Viral etiologies identified among RSV co-infection cases**

| **VIRAL PATHOGENS** | Viral load  (of the non-RSV viruses when co-infected with RSV) | Single infection load  (of the non-RSV viruses) | RSV viral load in co-infection cases |
| --- | --- | --- | --- |
|  | N=101 | N=174 | Log copies/ml Median (IQR) |
| Flu A, n(%) | 5 (5) | 5 (3) |  |
| Median Ct value flu A (IQR) | 33 (29-34) | 30 (30-31) | 6.7 (4.6-6.7) |
| Flu B, n(%) | 3 (3) | 3 (2) |  |
| Median Ct value flu B (IQR) | 30 (20-34) | 32 (29-33) | 7.2 (6.5-7.5) |
| AdV, n(%) | 18 (18) | 3 (2) |  |
| Median Ct value AdV (IQR) | 35 (33-36) | 36 (30-35) | 7.2 (6.3-7.8) |
| EnV, n(%) | 27 (27) | 2 (1) |  |
| Median Ct value EnV (IQR) | 34 (31-36) | 32 (29-35) | 7.6 (6.7-7.3) |
| MPV, n(%) | 4 (4) | 17 (10) |  |
| Median Ct value MPV (IQR) | 36 (33-38) | 26 (23-29) | 8.3 (8.3-8.5) |
| RV, n(%) | 45 (45) | 87 (50) |  |
| Median Ct value RV (IQR) | 30 (28-31) | 26 (24-28) | 7.5 (6.6-8.1) |
| PIV-1, n(%) | 0 | 5 (3) |  |
| Median Ct value PIV-1 (IQR) |  | 28 (26-30) | N.A |
| PIV-2, n(%) | 0 | 4 (2) |  |
| Median Ct value PIV-2 (IQR) |  | 22 (22-23) | N.A |
| PIV-3, n(%) | 5 (5) | 18 (10) |  |
| Median Ct value PIV-3 (IQR) | 37 (25-38) | 25 (23-32) | 6.2 (3.9-6.6) |
| PIV-4, n(%) | 2 (2) | 13 (7) |  |
| Median Ct value PIV-4 (IQR) | 35 (34-36) | 29 (25-33) | 7.6 (6.5-8.8) |
| CoV, n(%) | 6 (6) | 8 (5) |  |
| Median Ct value CoV (IQR) | 30 (28-33) | 27 (24-29) | 6.3 (4.6-6.5) |
| PeV, n(%) | 7 (7) | 2 (1) |  |
| Median Ct value PeV (IQR) | 29 (28-29) | 28 (26-31) | 7.3 (6.2-8.0) |
| BoV, n(%) | 11 (11) | 7 (4) |  |
| Median Ct value BoV (IQR) | 35 (26-37) | 23 (22-30) | 7.5 (6.9-7.9) |
